# Upregulation of Bcl-2 and Its Promoter Signals in CD4+ T Cells during Neuromyelitis Optica Remission

**DOI:** 10.3389/fnins.2017.00011

**Published:** 2017-01-24

**Authors:** Tao Yang, Su Wang, Xiao Yang, Qi Zheng, Lei Wang, Qian Li, Mingyan Wei, Zongpan Du, Yongping Fan

**Affiliations:** ^1^Department of Traditional Chinese Medicine, Beijing Tiantan Hospital, Capital Medical UniversityBeijing, China; ^2^Department of Tumor Radiotherapy, Hiser Medical Center of QingdaoQingdao, China; ^3^School of Management Science and Engineering, Shandong University of Finance and EconomicsJinan, China; ^4^Department of Oncology, Guang An Men Hospital of China Academy of Chinese Medical SciencesBeijing, China; ^5^School of Traditional Chinese Medicine, Capital Medical UniversityBeijing, China

**Keywords:** neuromyelitis optica, multiple sclerosis, Bcl-2, NFκB, MAP3K7

## Abstract

The homeostatic balance between production and elimination of CD4+ T cells in peripheral blood plays an important role in patients with neuromyelitis optica (NMO). The objective of the present study was to evaluate the anti-apoptosis genes Bcl-2 and its promoter signal (nuclear factor kappa-light-chain-enhancer of activated B cells, NFκB) in CD4+ T cells. Healthy subjects (HS, *n* = 25) and patients with multiple sclerosis (MS) (*n* = 25) and NMO (*n* = 30) in remission were consecutively enrolled in this prospective study between May and December 2015. CD4+ T cells were isolated using magnetic beads coated with anti-CD4 monoclonal antibodies, and gene expression of Bcl-2, NFκB, phosphatidylinositol-4, 5-bisphosphate 3-kinase/protein kinase B (PI3K/Akt), and MAP kinase kinase kinase 7 (MAP3K7) was measured by real-time reverse transcription-polymerase chain reaction (rt-PCR). Cytokines of tumor necrosis factor (TNF)-α and interleukin (IL)-1β were detected using human cytokine multiplex assay. Bcl-2 and NFκB gene expressions were elevated in NMO patients (1.63 ± 0.25; 2.35 ± 0.25) compared with those of HS (0.90 ± 0.11; 1.42 ± 0.22) and/or MS patients (1.03 ± 0.18; 1.55 ± 0.20) (*P* < 0.05). MAP3K7, but not Akt, was increased in NMO patients (1.23 ± 0.18; 1.56 ± 0.22) (*P* < 0.01) and was a significant factor related to elevated NFκB gene expressions (*P* < 0.001). On the other hand, IL-1β and TNF-α were also detected in the study and the results showed that both were elevated in NMO patients (23.84 ± 1.81; 56.40 ± 2.45) (*P* < 0.01; *P* < 0.05, respectively). We propose that MAP3K7 induced by IL-1β and TNF-α but not Akt promotes NFκB expression and, in turn, prolongs Bcl-2–mediated survival of CD4+ T cells in NMO patients.

## Introduction

**Figure 1:**
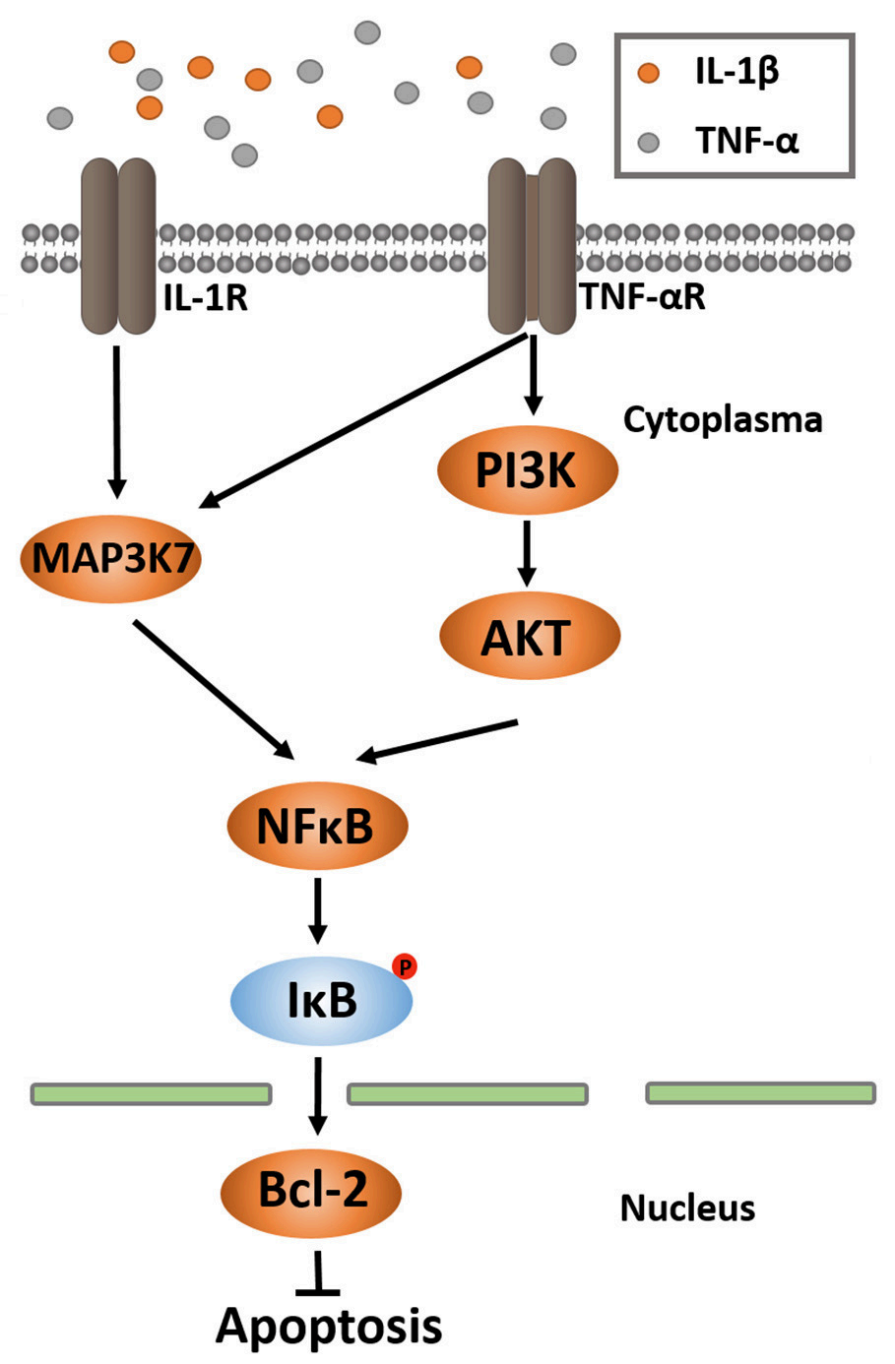
**MAP3K7 and PI3K/AKT pathways of NF-κB activation**. NF-κB activation occurs through many different pathways. Two of them are MAP3K and PI3K/AKT. In both ways, activated MAP3K7 and PI3K/AKT phosphorylate NF-κB, leading to IκB phosphorylation, ubiquitination and proteosomal degradation. A NF-κB dimer is released and translocated into nucleus, where it binds to the κB-binding site for upregulation of Bcl-2 gene expression. Bcl-2 executes the function of anti-apoptosis.

Neuromyelitis optica (NMO) is an inflammatory disease characterized by NMO immunoglobulin IgG (NMO-IgG), which is primarily considered to be an AQP4-specific antibody in NMO serum (Wingerchuk et al., 2015). Despite that B cells and auto-antibodies play decisive pathogenic roles in NMO, several lines of evidence suggest that T cells and cytokines have relevant roles in NMO (Varrin-Doyer et al., 2012; Zeka et al., 2015). Passive aquaporin (AQP) 4-IgG transfer alone does not produce central nervous system (CNS) pathology, but does promote the development of NMO-like lesions in recipient animals when CNS inflammation is induced by myelin-specific T cells (Vaknin-Dembinsky et al., 2012; Varrin-Doyer et al., 2012). This indicates that AQP4-IgG–specific CD4+ T cells participates in the genesis of NMO and that T cells appear to be equally crucial for the full development of the immunopathogenetic cascade (Vaknin-Dembinsky et al., 2012). Previous studies have identified the myelin/MOG specific T cells to be CD4+ cells in spontaneous CNS autoimmunity and NMO like diseases (Dasgupta and Dasgupta, 2016; Klotz et al., 2016; Tostanoski et al., 2016). Once the T cell is stimulated by agents such as TNF-α and IL-1β, the phosphorylated MAP3K7 could phosphorylate IκB, which has the ability to phosphorylate NFκB (DiDonato et al., 1997). And, NFκB signals are required to promote the expression of Bcl-2. Protein kinase B (Akt) was suggested in another study to be an important driver of the glycolytic phenotype and is linked to many cellular processes, including anti-apoptosis and anti-autophagy (Majumder et al., 2004). Bcl-2 and its family members have the ability to control apoptosis and anti-apoptosis in balancing mitochondria permeability (the most common form of programmed cell death in biology and disease; Hotchkiss et al., 2009; Figure 1). In NMO and multiple sclerosis (MS) patients and their animal models, subtypes of CD4+ T cells were elevated compared to those of HS, and we found that one corollary of these events is the lack of balance between apoptosis and anti-apoptosis in CD4+ T cells (Uzawa et al., 2010; Wang et al., 2011). This indicates that the homeostatic balance between production and elimination of CD4+ T cells in peripheral blood plays a special role in NMO. Even though a number of pathological or physiological processes contributing to anti-apoptosis have been described, limited understanding of anti-apoptosis in CD4+ T cells in NMO hinders the development of an effective treatment. CD4+ T cell subpopulations accumulate in peripheral blood mononuclear cells (PBMCs) in NMO patients, implying anti-apoptosis might be involved in pathogenesis. Bcl-2, which has anti-apoptosis properties, was designed to value in this study. The objective of the current study was to analyze the anti-apoptotic factors in CD4+ T cells, such as Bcl-2 and its promoter signals, in NMO patients during clinical remission. We also evaluated the effects of cytokines on CD4+ T cell anti-apoptosis.

## Patients and methods

### Study populations

Written informed consent was obtained from all subjects. The current study protocol was approved by the local ethics committee of Beijing Tiantan Hospital Affiliated with Capital Medical University China (No. KY2015-003-02). The study population comprised MS and NMO patients who were recruited from Beijing Tiantan Hospital (age 16–60; all patients met NMO or MS diagnosis; without other immune diseases; written informed consent was obtained) and healthy subjects (HS) (age 16–60; without immune diseases) from the general population. Infections were ruled out by full blood count in all subjects. This study was conducted between May and December 2015 on 25 HS, 30 MS patients, and 30 NMO patients. The NMO diagnosis was based on the revised diagnostic criteria (2006) for NMO (Wingerchuk et al., 2006; the 2015 diagnostic criteria had not been published when the study was carried out) and the MS diagnosis was determined according to the 2010 revised McDonald criteria (Polman et al., 2011).

### CD4+ T cells collection

Peripheral blood were obtain at 9:00 a.m. using disposable ethylenediaminetetraacetic acid (EDTA) vacuum blood collection tubes (BD, USA), and PBMCs were collected by centrifugation on a Ficoll-Hypaque density gradient. The purified CD4+ T cells were isolated using magnetic beads coated with anti-CD4 monoclonal antibody (Miltenyi Biotec, Germany) following the manufacturer's instructions.

### Quantitative real-time polymerase chain reaction (qRT-PCR)

Total RNA was isolated from approximately 50 to 100 mg purified CD4+ T cells using Trizol. The concentration of each RNA sample was measured spectrophotometrically. The integrity of RNA samples was assessed by agarose gel electrophoresis. The cDNA was synthesized using the PrimeScript™ RT reagent Kit with gDNA Eraser (1.0 μL) (TaKaRa bio, Japan). The PCR primers were designed using Primer Premier 6.0 (http://www.premierbiosoft.com/primerdesign/) based on the GenBank Accession codes and synthesized in Invitrogen (China). Quantitative measurement of mRNA was performed using the ABI Prism 7500 (Applied Biosystems, USA). These data were analyzed using the comparative 2^−ΔΔCT^ method (Livak and Schmittgen, 2001).

### Plasma cytokines levels

After standing for 2.0 h at 4.0°C, the supernatant of blood samples was pipetted into EP tubes and stored at −80°C. Plasma IL-1β and TNF-α were measured using MILLIPLEX® MAP Human High Sensitivity Cytokine Panels (Cat. No. HCYTOMAG-60K) according to the manufacturer's instructions.

### Statistical analysis

Statistical analysis was performed using GraphPad Prism version 5 (GraphPad Software, Inc., California, USA) and the data were reported as box and Whisker plots with the Whiskers representing minimum and maximum values. Multi-group comparisons of the means with one independent variable were carried out by one-way analysis of variance (ANOVA) test with *post hoc* contrasts by Student-Newman-Keuls test. The *t*-test was used to compare 2 groups. Pearson's test was used to perform correlations. Power analysis was performed using G^*^Power (Version:3.1, Universität Regensburg, Berlin, German). A *P* < 0.05 was considered statistically significant.

## Results

We identified 25 HS, 25 MS patients, and 30 NMO patients according to demographic and clinical data (Tables 1, 2). NMO patients had more severe clinical neurological defects than MS patients (*P* < 0.01) (Table 1).

**Table 1:** **Demographic and clinical data of healthy subjects (HS) and multiple sclerosis (MS) and neuromyelitis optica (NMO) patients**.

		**HS (*n* = 25)**	**MS (*n* = 25)**	**NMO (*n* = 30)**
Gender	F/M	18/7	18/7	25/5
Age (year)	Range	16–54	16–53	13–55
	Mean±SE	34.84 ± 2.24	33.48 ± 2.15	35.43 ± 2.28
Age at onset (year)	Range	–	6–51	11–53
	Mean±SE	–	29.60 ± 2.18	29.37 ± 2.14
Disease duration (year)	Range	–	0.25–11.92	0.17–21.50
	Mean±SE	–	3.91 ± 0.53	5.58 ± 0.87
ARR	Range	–	0.29–8.00	0.09–12.00
	Mean±SE	–	1.48 ± 0.32	1.69 ± 0.41
EDSS	Range	–	0–5.0	1–6.5
	Mean±SE	–	1.92 ± 1.39	3.07 ± 0.31^**^

*HS, healthy subjects, MS, multiple sclerosis, NMO, neuromyelitis optica, EDSS, expanded disability status scales; ARR, annual relapse rate*.

** *P < 0.01*.

**Table 2:** **Details of MS and NMO patients**.

**Patient No**.	**Age (year)/gender**	**Disease dur (month)**	**Dur to the last relapse**	**EDSS**	**No. of relapse**
MS-01	26/M	23	25	2.5	3
MS-02	53/M	51	20	2.5	3
MS-03	45/F	40	57	0	3
MS-04	33/F	30	33	1.5	3
MS-05	39/F	38	7	3	2
MS-06	32/M	27	59	0	3
MS-07	48/F	42	68	2.5	2
MS-08	18/F	14	44	3.5	2
MS-09	26/F	24	18	0	3
MS-10	49/F	45	48	2	2
MS-11	25/F	20	59	2.5	10
MS-12	24/M	21	39	0	4
MS-13	34/F	31	42	1	1
MS-14	16/F	14	21	1.5	6
MS-15	46/F	39	85	1	5
MS-16	25/M	23	22	3	2
MS-17	40/M	34	75	2	2
MS-18	42/F	41	16	2	3
MS-19	24/M	24	3	3.5	2
MS-20	42/F	39	43	3.5	5
MS-21	29/F	24	67	3	2
MS-22	34/F	31	34	2.5	1
MS-23	44/F	36	102	0	7
MS-24	26/F	23	44	5	2
MS-25	17/F	6	143	0	5
NMO-01	24/F	15	114	3	7
NMO-02	20/F	16	55	1.5	4
NMO-03	30/F	22	95	2	6
NMO-04	55/F	53	30	5.5	3
NMO-05	22/F	19	38	3.5	4
NMO-06	14/M	13	18	6.5	5
NMO-07	51/F	48	43	3	6
NMO-08	36/F	24	150	1.5	6
NMO-09	46/F	37	108	2.5	3
NMO-10	26/M	22	40	1	6
NMO-11	55/F	47	88	6.5	5
NMO-12	50/F	35	180	1.5	7
NMO-13	19/M	16	30	1.5	4
NMO-14	34/F	22	15	3.5	5
NMO-15	31/F	24	82	3.5	8
NMO-16	43/M	36	107	1	3
NMO-17	37/F	31	71	3.5	6
NMO-18	29/F	27	14	2	3
NMO-19	39/F	29	122	6	4
NMO-20	34/M	30	44	6	4
NMO-21	53/F	29	258	5.5	2
NMO-22	32/F	26	73	3	4
NMO-23	40/F	40	2	2.5	2
NMO-24	52/F	50	23	3	3
NMO-25	48/F	45	13	2	2
NMO-26	24/F	20	46	3	4
NMO-27	38/F	31	83	3.5	5
NMO-28	23/F	21	23	1	3
NMO-29	13/F	11	15	1.5	6
NMO-30	45/F	42	29	2	4

*No, number; F, female; M, male; EDSS, expanded disability status scales; Dur, duration*.

**Figure 2:**
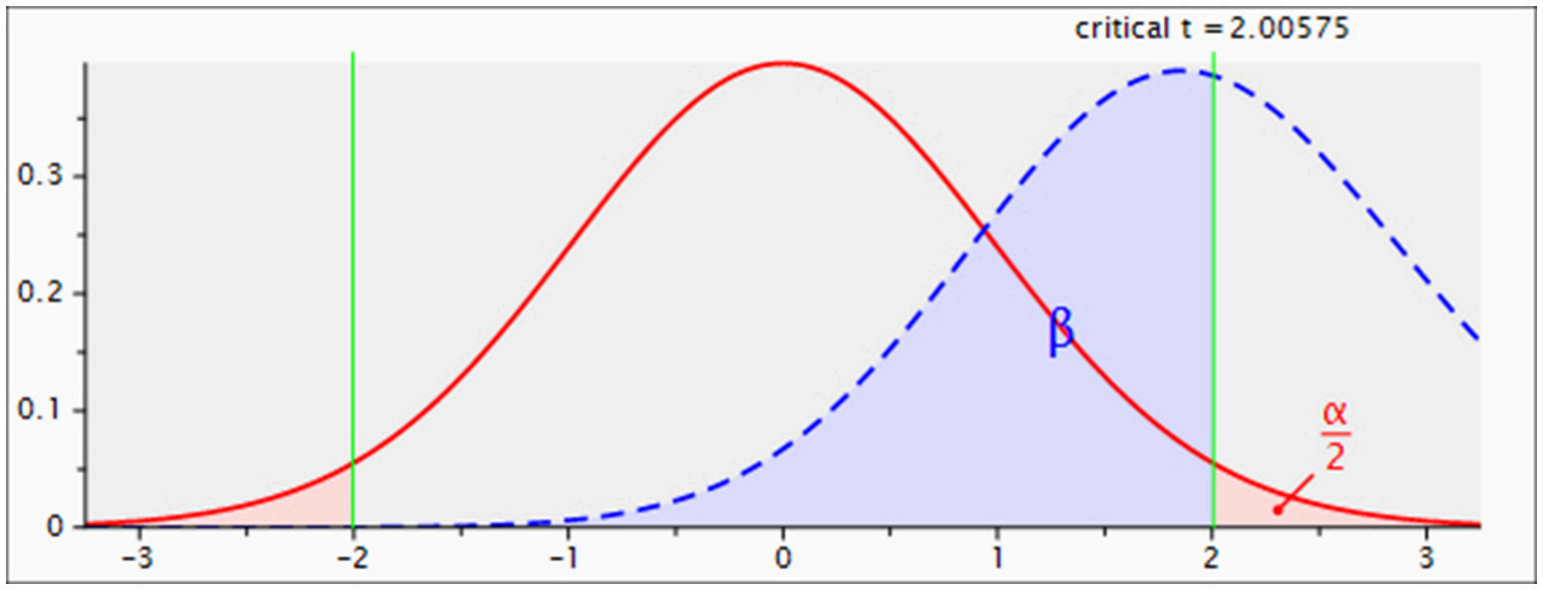
**Power analysis of Bcl-2 gene expressions between MS and NMO patients**. Setting parameter: *n*_1_ = 25, *n*_2_ = 30, results: power = 0.46. n_1_: number of MS patients; n_2_: number of NMO patients.

**Figure 3:**
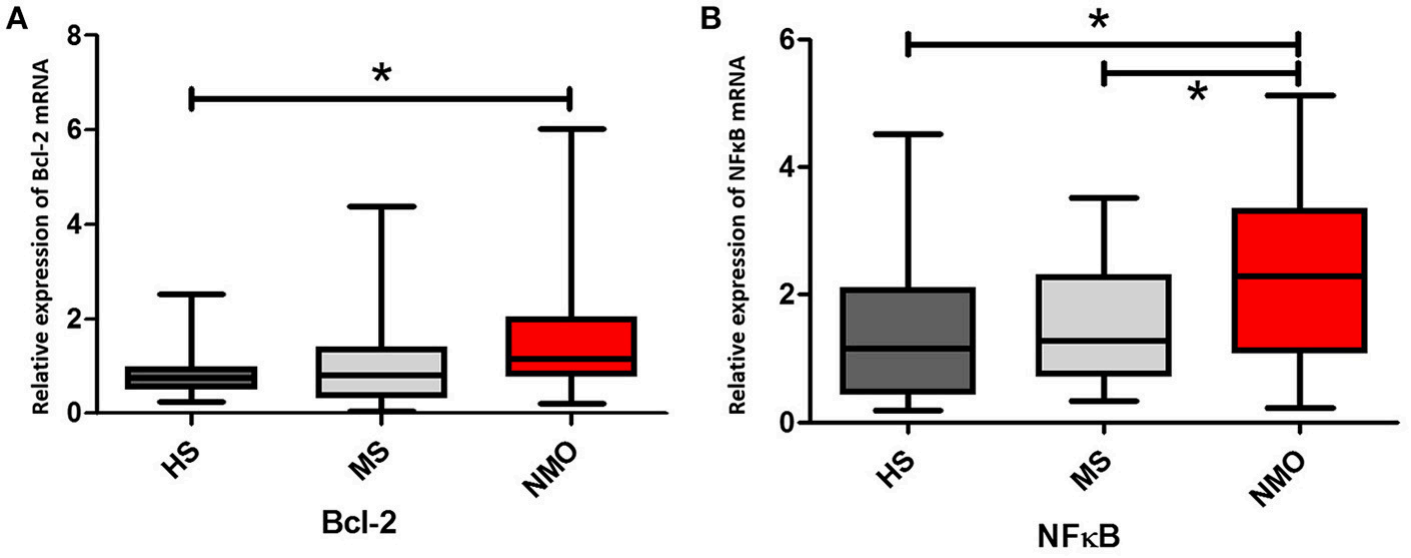
**Bcl-2 and NFκB genes expressions in CD4+ cells with HS, MS and NMO patients**. **(A)** Comparison of Bcl-2 mRNA expression in CD4+ cells with HS, MS and NMO patients. **(B)** Comparison of NFκB mRNA expression in CD4+ cells with HS, MS and NMO patients. HS = healthy subjects, MS = multiple sclerosis, NMO = neuromyelitis optica. ^*^P < 0.05.

We initially investigated the Bcl-2 and NFκB gene expressions in the CD4+ T cells of HS, MS patients, and NMO patients. Overexpression of anti-apoptotic member Bcl-2 in NMO patients conforms to the higher NFκB expression in those patients (**Figure 3A**). NMO patients had higher expression of Bcl-2 gene comparing with HS (*P* < 0.05). Even though the results indicated NMO patients (mean = 1.63) had higher expression of Bcl-2 than MS patients (mean = 1.03), there were non-significant differences between the two (*P* = 0.06). The power analysis suggested this result might be caused by the limited number of recruiting members (setting parameter: *n*_1_ = 25, *n*_2_ = 30, results: power = 0.46; Figure 2). And, suppose power = 0.8 and 0.5 are satisfied, *n* = 62 and 31 in each group respectively. Given that NFκB signals are required to promote the expression of Bcl-2 gene, we examined NFκB gene expression (Wang et al., 2015). NMO patients had higher NFκB expression than HS and MS patients (*P* < 0.05) (Figure 3B).

**Figure 4:**
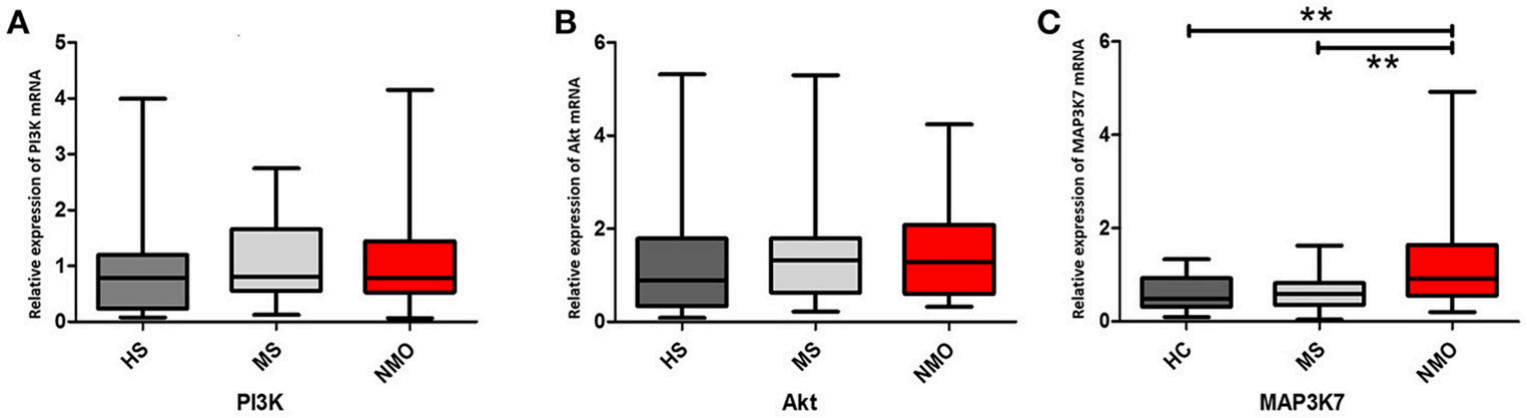
**PI3K/Akt and MAP3K7 genes expressions in CD4+ cells with HS, MS and NMO patients**. **(A)** Comparison of PI3K mRNA expression in CD4+ cells with HS, MS and NMO patients. **(B)** Comparison of Akt mRNA expression in CD4+ cells with HS, MS and NMO patients. **(C)** Comparison of MAP3K7 mRNA expression in CD4+ cells with HS, MS and NMO patients. HS = healthy subjects, MS = multiple sclerosis, NMO = neuromyelitis optica. ^**^P < 0.01.

Phosphatidylinositol-4, 5-bisphosphate 3-kinase (PI3K) and Akt genes were also tested in this study. Although PI3K and Akt enhances Bcl-2 promoter activity by phosphorylating NFκB, which is elevated in NMO patients, PI3K and Akt showed no significant increase in NMO patients (*P* > 0.05) (Figures 4A,B). Lacking differences in PI3K and Akt expressions in NMO patients, MAP kinase kinase kinase 7 (MAP3K7), another NFκB promoter, was measured. The results showed a significant increase in the expression of MAP3K7 in NMO patients (vs. HS, *P* < 0.05; vs. MS patients, *P* < 0.05) (Figure 4C).

**Figure 5:**
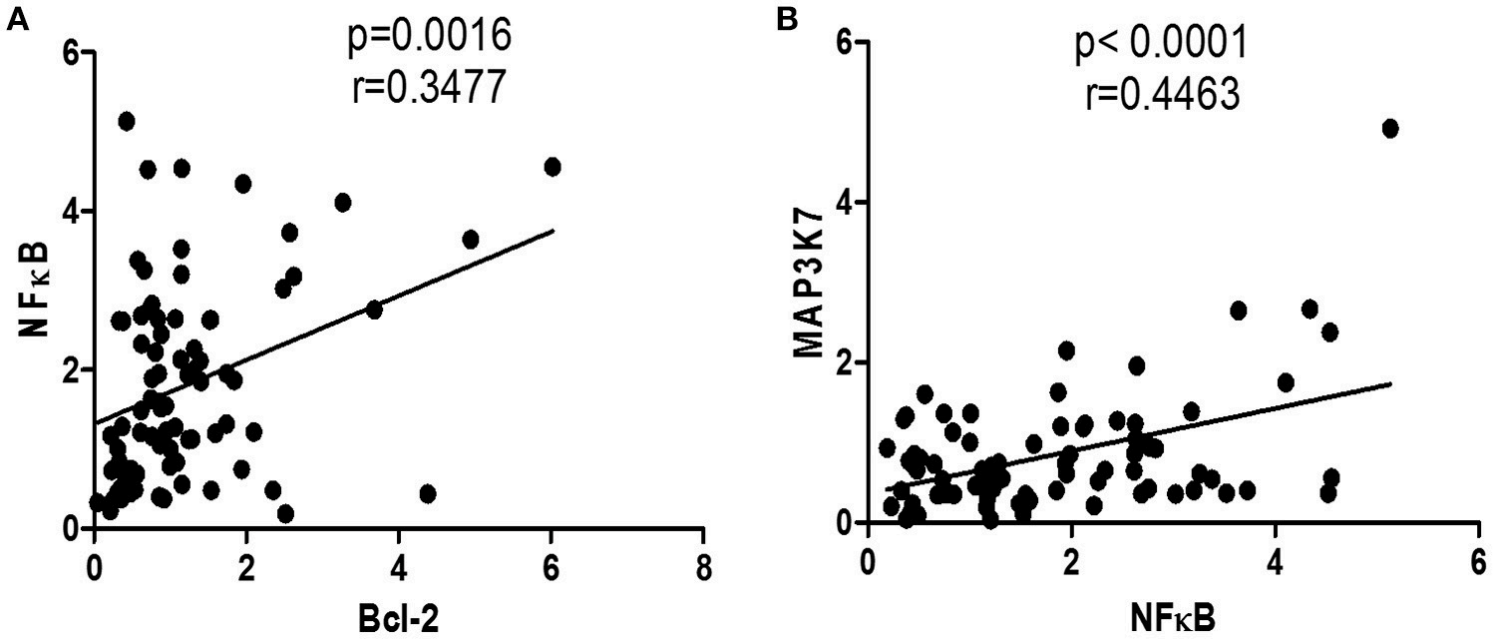
**Correlation of gene expressions of all subjects**. **(A)** Correlation between Bcl-2 and NFκB mRNA expressions in all subjects. **(B)** Correlation between NFκB and MAP3K7 mRNA expressions in all subjects.

Given that upregulating MAP3K could promote NFκB gene expression, which, in turn, could enhance Bcl-2 gene expression, the correlations between NFκB and Bcl-2 and between MAP3K7 and NFκB were analyzed. Bcl-2 and NFκB and NFκB and MAP3K7 had significant correlations among all subjects (*P* = 0.0016, *P* < 0.0001, respectively; Figures 5A,B).

**Figure 6:**
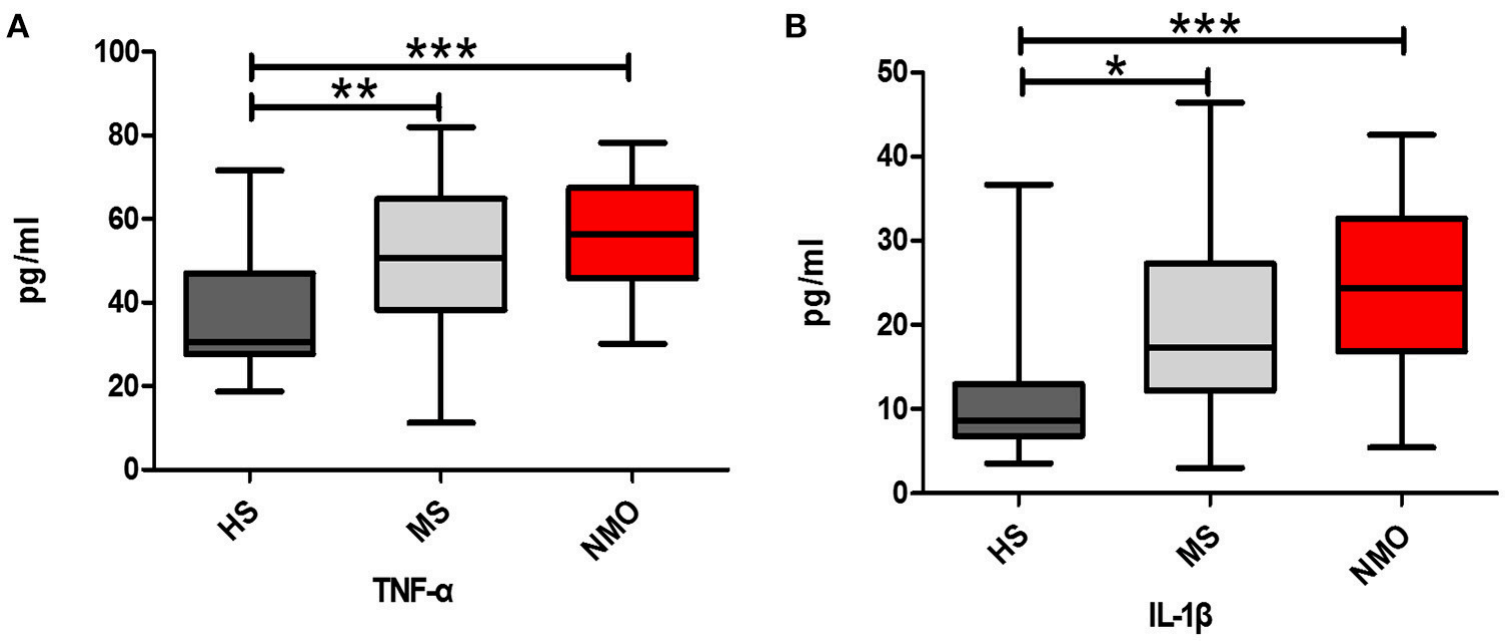
**Comparison of cytokine levels among HS, MS and NMO patients**. **(A)** Comparison of plasma TNF-α levels among the HS, MS and NMO. **(B)** Comparison of plasma IL-1β levels among the HS, MS and NMO. HS = healthy subjects, MS = multiple sclerosis, NMO = neuromyelitis optica. ^*^P < 0.05, ^**^P < 0.01, ^***^P < 0.001.

The cytokines TNF-α and IL-1β were detected in the study. NMO patients have higher TNF-αlevels compared to HS (*P* < 0.05) and NMO patients, and MS patients had higher levels of IL-1β compared to HS (*P* < 0.01, *P* < 0.05, respectively; Figures 6A,B).

## Discussion

Unlike MS patients, NMO patients have a more severe CNS demyelinating syndrome characterized by bilateral simultaneous neuritis and acute myelitis which is in accordance with the Expanded Disability Status Scale we observed for these patients (O'Connor et al., 1998; Huh et al., 2014; Wingerchuk et al., 2015). Passive transfer of IgGs from NMO patients to experimental autoimmune encephalomyelitis rats reduced the expression of AQP4 in astrocytes coupling with severer clinical neurological functions (Bennett et al., 2009; Bradl et al., 2009). Conversely, unimmunized rats with IgGs from NMO patients showed a lack of pathology and neurological dysfunction, suggesting that T cells, complements, and leukocytes participate in the pathogenesis of AQP4-Ab–mediated pathology in NMO patients (Chihara et al., 2011). Dysregulation in the cell death of T cells leads to prolonged activation of autoreactive T cells (Hagman et al., 2015). Bcl-2, which was originally identified in follicular B-cell lymphomas and localized to the cytoplasmic face of intracellular membranes (outer mitochondrial membrane, endoplasmic reticulum), inhibits apoptosis (Marsden and Strasser, 2003). Considering that the Bcl-2 has the ability to prolong cell survival, CD4+ T cells can live longer in NMO patients and might accumulate in peripheral blood to cause more severe clinical features. In the current study, we analyzed Bcl-2 gene expression in HS, MS patients, and NMO patients. We found that Bcl-2 gene expression in CD4+ T cells of NMO patients was higher than that in HS (*P* < 0.05). Based on the Bcl-2-mediated dual purpose of inhibiting apoptosis and autophagy (Sasi et al., 2009; Scarfò and Ghia, 2013), this accumulation of CD4+ T cells indicates that Bcl-2 might play a critical role in anti-apoptosis and anti-autophagy by modulating the permeability of the mitochondria in NMO patients. This study showed Bcl-2 and its promoter signals as NFκB, MAP3K7, TNF-α, and IL-1β were upregulated in CD4+ T cells during NMO remission. Azathioprine-reduced Bcl-2 synthesis and release could lead to mitochondrial dysfunction and cell death, as suggested an effective treatment for NMO (Menor et al., 2004; Papadopoulos et al., 2014).

In addition to Bcl-2 expression upregulated in NMO patients, NFκB and its pathway were measured in this study. The NFκB pathway plays an important role in inflammation, immunity, apoptosis, and autophagy by regulating several immunoregulatory factors (Verma and Manna, 2016). It is also involved in both immune cell development and function, and is critical in modulating immune response through transcriptional regulation of cytokines and chemokines. The NFκB family of transcription factors has been shown to regulate various aspects of T cell development, activation, differentiation, and survival (Oh and Ghosh, 2013). NFκB1 is one of the major targets of the canonical pathway and the results in the current study showed that NMO patients had higher NFκB 1 gene expression compared with HS and MS patients (*P* < 0.05). Notably, Bcl-2 gene expression had a significant correlation with NFκB1 gene expression in all test subjects (*P* = 0.0016). This illumined NFκB1 pathway has an important role in increasing Bcl-2 gene expression, in turn, to prolong the CD4+ T cells that might participate in pathogenesis in NMO patients.

Other works have shown that activated PI3K and MAP3K7 results in phosphorylating Akt, which, in turn, upregulates Bcl-2 by enhancing Bcl-2 promoter activity (Pugazhenthi et al., 2000). The current study showed that the PI3K/Akt pathway had not changed in the NMO patients compared to that in MS patients and HS, which indicated that the PI3K/Akt pathway might not play a role in NMO pathogenesis (*P* > 0.05). Meanwhile, MAP3K7 gene expression increased in NMO patients compared to that in HS and MS patients (*P* < 0.01). To analyze whether the expression of NFκB1 is associated with the conversion of MAP3K7, the correlation among them was tested. The results showed that MAP3K7 is strongly correlated with NFκB1 (*P* < 0.0001). This indicated that MAP3K7 has a decisive role in upregulating NFκB1 rather than PI3K/Akt in NMO patients.

Usually, NFκB1 is sequestered in the cytoplasm of resting cells by association with IκB family proteins (Karin and Ben-Neriah, 2000). Once the cell is stimulated by agents such as TNF-α and IL-1β, NFκB1 is activated through MAP3K7 phosphorylation (DiDonato et al., 1997). TNF-α and IL-1β have the ability to transmit survival signals as Bcl-2 by activating MAP3K7/NFκB1 transcription factors (Hagman et al., 2015). In this study, both were evaluated in NMO patients (vs. HS, *P* < 0.001) and MS patients (vs. HS, *P* < 0.05). These factors, such as Bcl-2, NFκB, MAP3K7, TNF-α, and IL-1β, somewhat inhibit apoptosis of CD4+ T cells, which might then interact with B cells and afford an opportunity to clinically relapse in NMO patients.

Collectively, this study indicated that MAP3K7 expression is stimulated by TNF-α and IL-1β and, in turn, activates NFκB 1, which has the ability to prolong Bcl-2-mediated CD4+ T cell survival in NMO patients. The aberrant longevity of CD4+ T cells can lead to pathological immune cell accumulation in NMO patients, which augments the risk of NMO relapse. These results might help identify clinical applications for enhancing the treatment of NMO; however, the study was not restricted to subgroups of CD4+ T cells and, thus, we need to test which kinds of CD4+ T cells have aberrant Bcl-2 and its promotor expressions in further studies, which might explain why MS patients have elevated subtypes of CD4+ T cells but with relatively regular Bcl-2 expression.

## Author contributions

TY drafted this manuscript. TY, SW, and LW performed the MILLIPLEX®; MAP Human High Sensitivity Cytokine/Chemokine Panels tests, performed the real-time rtPCR, and participated in collecting samples. LW, XY, QZ, QL, MW, and ZD performed the statistical analysis and evaluated the EDSS scores of patients. TY, SW, and YF participated in the design and coordination of the study. TY conceived of the study and participated in its design. LW revised the manuscript. All authors read and approved the final manuscript.

### Conflict of interest statement

The authors declare that the research was conducted in the absence of any commercial or financial relationships that could be construed as a potential conflict of interest.
